# Decline in lumbar extensor muscle strength the older adults: correlation with age, gender and spine morphology

**DOI:** 10.1186/1471-2474-14-215

**Published:** 2013-07-22

**Authors:** Devinder Kaur Ajit Singh, Martin Bailey, Raymond Lee

**Affiliations:** 1 Faculty of Health Sciences, Jalan Raja Muda Aziz, Universiti Kebangsaan Malaysia, Kuala Lumpur 50300, Malaysia; 2 Chelsea School, University of Brighton, Hillbrow Denton Road, Eastbourne BN20 7SR, UK; 3 Roehampton University, Roehampton Lane, SW15 5PU, London, UK

**Keywords:** Lumbar extensor muscle strength, Thoracolumbar curvatures, Muscle fibre angles

## Abstract

**Background:**

Muscle morphology, age and gender may be determinants of muscle strength in older adults. However, very few research studies have directly examined such correlation in the ageing spine. The aim of the study was to examine the correlation between lumbar extensor muscle strength, its muscle fibre angles, thoracolumbar curvature, age and gender in the older and younger adults.

**Methods:**

Muscle fibre angles of lumbar extensor muscles, thoracolumbar curvature and lumbar extensor muscle strength were examined in 26 young (mean age 27.9, SD 5.2) and 26 older (mean age 72.1, SD 5.9) participants. Pearson’s correlation was employed to determine the association among lumbar extensor muscle fibre angle, thoracolumbar curvature, age, gender and lumbar extensor muscle strength. Multiple stepwise linear regression analysis was used to identify significant determinants of lumbar extensor muscle strength.

**Results:**

The results demonstrated a significant correlation between lumbar extensor muscle strength, muscle fibre angle, age and gender. In the step wise regression analysis, both gender and age were identified as the most robust determinant for lumbar extensor muscle strength in older adults. However, gender was the only determinant of muscle strength in the young.

**Conclusion:**

These results suggest that the decline in the lumbar extensor muscle strength in older adults was more dependent on age when compared to younger adults.

## Background

Muscle strength declines and accelerates further with age [1,2]. Previous studies recorded an approximately 50% decline in lumbar extensor muscle strength from the third to sixth decade of life [3]. Trunk extensor muscle strength is greater in males and reported to decline more with ageing in males [4]. Both age and gender have also been found to be correlated with trunk extensor muscle strength in previous studies of different groups of participants [5,6]. Poor physical performance; increased risk of falls, fractures and disability; increased mortality and poor quality of life; fear induced inactivity are associated with a decrease in muscle strength in older adults [7-12].

Alterations and adaptations in lumbar spine morphology that includes lumbar extensor muscle fibre angles [13] and thoracolumbar curvatures [14] may lead to deterioration in lumbar extensor muscle strength [15]. Previous studies have examined the inter relationship between trunk extensor muscle strength with anthropometric measurements such as age, gender, body mass index, upper and lower limb strength together with psychosocial factors and predicting the trunk extensor musculature strength from these variables [4,6,16,17]. Lumbar extensor muscle fibre angles and thoracolumbar curvatures changes in ageing may interact with age and gender. However, no study has been directed to examine this and at predicting lumbar extensor muscle strength from these morphological indices. It is clinically important to examine the relationship between lumbar extensor muscle strength, lumbar extensor muscles fibre angles and thoracolumbar curvature since this relates to mechanisms of muscle force production and may help to explain how muscle function deteriorates with age in the spine.

Lumbar extensor muscle strength is dependent on many factors [18] and accurate strength prediction so far has not been completely accounted for. In addition, predictors of trunk extensor muscle strength have either been carried out for a younger group or on patients with low back pain. Some of the indices used to predict trunk extensor muscle strength presently such as body mass index and body circumference may change in older adults so the prediction equation may become inaccurate for use with older adults.

Lumbar musculature strength is also desirable if a deficiency from the normal is to be recognised in older adults. Furthermore, it is important for the assessment of functional performance capacity of older adults in daily living activities. However, performing lumbar strength measurements presents some challenges. Lumbar extensor muscle strength testing poses difficulty with muscle isolation and fixation. Occasionally, it cannot be performed because the physical health of older adults does not allow it and their motivation may be affected. Hence, it will be useful to approximate lumbar extensor muscle strength of older adults from a regression model.

The aim of this study was to examine the correlation between lumbar extensor muscle strength, lumbar extensor muscle fibre angles, thoracolumbar curvature, age and gender in the older and younger adults.

## Methods

### Participants

Fifty two participants (26 younger participants aged 20 to 35 and 26 older participants, aged 65 to 84) participated in the study from a local community area. Prior to the tests, participants were assessed against the following exclusion criteria: had any history of current back pain, or back pain requiring medical attention in the last 6 months; serious trauma leading to fractures or dislocations of the spine; prior surgery to the back; x-ray of the spine revealing underlying pathologies such as tumours, spinal infections, tuberculosis; any known inflammatory joint diseases, rheumatological conditions, spinal deformities such as scoliosis, spondylolisthesis, spondylolisis; any neurological deficits, and were taking prescribed drugs that potentially affect muscle strength, such as corticosteroids. Ethical approval was obtained from the Faculty Research Ethics and Governance Committee of The University, and informed written consent was obtained from all participants.

### Lumbar extensor muscle fibre angles

Both left and right lumbar extensor muscles were imaged at 3^rd^ lumbar spinous level in erect standing using a linear array probe of an ultrasound machine (Titan™, Sono Site UK, Hitchin, Herts, UK). Saved captured images were then analysed using Matlab (Version 1.9.1 MathWorks, Inc., Natrick, MA, USA). Muscle fibre angles of mid-substance of the muscles were determined. Details of the procedures were as presented in our earlier study [13].

### Thoracolumbar curvature

This procedure has been reported in Singh et al. (2010) [14]. Briefly, measurements of thoracolumbar curvature were performed with an electromagnetic tracking device (Fastrak Polhemus, 40 Hercules Dr, Colchester, VT 05446) by tracing the whole spinous processes of the thoracolumbar spine. Both the left and right posterior iliac spine; 1^st^ and 8th thoracic; 1^st^ and 5^th^ lumbar spinous levels were digitized. The thoracolumbar curvature angles were determined using a pre written software using Matlab (Version 1.9.1 MathWorks, Inc., Natrick, MA, USA).

### Lumbar extensor muscle strength

Lumbar extensor muscle strength was measured using a load cell (LCM 200, 1112N; Futex Advanced Sensor Technology, Inc., Irvine, California), connected to the upper trunk with the participants standing in a restraining frame. Participants did three five seconds sustained maximum voluntary contraction (MVC) with a 5 minutes rest in between. Muscle strength was calculated as moments generated by the product of highest force MVC value with the moment arm. This procedure has been reported in our earlier study [15].

### Statistical analysis

Data were analysed using SPSS software version 15 (SPSS Inc. Chicago, USA). Preliminary analysis of the data showed that the data was normally distributed and scatter plots suggested linear relationships. Levene’s test for homogeneity of variances showed no significant differences (p > 0.05) in lumbar extensor muscle fibre angles, thoracolumbar curvature and lumbar extensor muscle strength. This result indicates that the differences in the two groups were equal. The differences in age, body mass index and physical activity score between the two groups were compared using student’s t-test. Correlation between lumbar extensor muscle strength, lumbar extensor muscle fibre angles, thoracolumbar curvature, age and gender were established with 2 tailed Pearson correlations. Age and gender were chosen to be included as these factors have been demonstrated in the literature to be important. Body mass index (BMI) was included in the regression to account for the potential effect of BMI on lumbar extensor muscle strength. Stepwise multiple linear regression analysis was used to identify significant predictors of lumbar extensor muscle strength, using lumbar extensor muscle strength as the dependent variable and lumbar extensor fibre angles, thoracolumbar curvature, BMI, age and gender as independent variables. The model was checked for multicollinearity problems using the tolerance (proportion of variability not explained) and VIF (variance inflation factor) values. A tolerance value of less than .10 and a VIF value that is above 10, indicates multicollinearity problems in the model [19].

## Results

The younger group were aged from 20 to 35 (10 males, 16 females), and the older group were aged 65 and above (10 males, 16 females). The mean age and associated standard deviations (SD) of the younger and older age groups were 27.9 (SD 5.2) and 72.1 (SD 5.9) respectively. The older age group had a mean BMI that was 1.6 kg/m^2^ higher than the younger age group which was determined to be a statistically significant difference (p = 0.03).

### Correlations

The combined results of 2 tailed Pearson correlations for both older and younger adults were as presented in Table 1. Age was negatively correlated with lumbar extensor muscle strength. Lumbar extensor muscle strength was positively associated with fibre angles and significantly larger in males. Similarly, the relationship between lumbar extensor muscle strength and the thoracic kyphosis was low and non significant. These results indicate that lumbar extensor muscle strength and fibre angles decrease with age. These results also suggest that lumbar trunk extensor muscle strength were greater with larger muscle fibre angles and in males.

**Table 1:** The combined results of 2 tailed Pearson correlations (r) between lumbar extensor fibre angles, lumbar moments, thoracolumbar curvature, age and gender for both older and younger adults

	**Age**	**Lumbar moment**	**Fibre angles**	**Thoracic kyphosis**	**Lumbar lordosis**	**Gender 1 = male, 2 = female**
Age	1	-.45**	-.50**	.38**	-.21	-.01
Lumbar moments	-	1	.40**	-.19	-.07	-.49**
Fibre angles	-	-	1	-.17	.03	-.24
Thoracic kyphosis	-	-	-	1	.15	.12
Lumbar lordosis	-	-	-	-	1	.38**
Gender	-	-	-	-	-	1
1 = male,						
2 = female						

** = p < 0.01.

### Multiple linear regression

BMI did not appear to be a predictor for lumbar extensor muscles strength for both younger and older adults. As the factors that can be fitted into a regression equation are limited based on the sample size, BMI was not included in the final regression analysis. The stepwise multiple linear regression analysis results for the younger and older group are as shown in Table 2. The stepwise multiple linear regression analysis indicated that in older adults, only age and gender were selected as prediction variable of lumbar extensor muscle strength. Gender accounted for 53% of the variance, whilst gender and age combined accounted for 64% of the variance in the lumbar extensor muscle strength. For the younger adults, lumbar extensor muscle strength was only significantly predicted by gender which accounted for 21% of the variance. The standardized beta value (β) for gender is higher in the model for older adults (−.68) compared to younger adults (−.46), indicating that gender is a more important variable in strength prediction for older adults. In comparison to gender, age (β = −.34) has a lower degree of importance in the model for older adults. Age does not appear to be a variable in the lumbar extensor muscle strength prediction model for younger adults.

**Table 2:** Stepwise multiple linear regression analysis of the significant independent variables on lumbar moment

	**Younger (10 males, 16 females)**				**Older (10 males, 16 females)**			
	**B**	**SE B**	**β**	**SEE**	**B**	**SE B**	**β**	**SEE**
Step 1								
Constant	85.21	16.31		23.98	63.75	7.81		11.48
Gender	−24.23	9.67	-.46*		−23.87	4.63	-.73***	
Step 2								
Constant	-	-	-		130.19	25.19		10.18
						4.14	-.68***	
Gender					−22.45	0.35	-.34*	
Age					−0.95			

Note: younger R^2^ = .21, older R^2^ = .53 for step 1, Δ R^2^ = .64.

*p < 0.05, ***p < 0.001.

Thus, the regression equations established were:

For the older adults:

Lumbar extensor muscles strength (Nm) = 130.19 - 22.45 × gender (1 = male, 2 = female) – 0.95 × age

For the younger adults:

Lumbar extensor muscles strength (Nm) = 85.21 - 24.23 × gender (1 = male, 2 = female)

The regression model, as predicted by the stepwise procedure, is shown in Table 3. The tolerance and VIF values in the present model indicated that there was no multicollinearity problem. The shrinkage of adjusted R^2^ from the actual R^2^ was small, indicating that there were not an excessive number of variables in the model and that the sample size was sufficient.

**Table 3:** Summary of the regression model

**Variables in equation**	**Younger (10 males, 16 females)**			**Older (10 males, 16 females)**		
	**Adjusted R**^**2**^	**Tolerance**	**VIF**	**Adjusted R**^**2**^	**Tolerance**	**VIF**
Gender	.17	1.00	1.00	.52	1.00	1.00
Age				.61	.98	1.01

Tolerance (proportion of variability not explained).

VIF (variance inflation factor values).

## Discussion

It is well documented in the literature that trunk muscle strength decreases in older adults [3,5,20]. However, the correlation between the decline in strength and spine morphology such as muscle fibre angles and thoracolumbar curvatures has not been examined adequately, especially in the older adults.

Thoracic kyphosis and lumbar lordosis angles showed only small inverse correlations with lumbar muscle strength which were not significant. The correlation of trunk extensor muscle strength with thoracic kyphosis in the earlier studies has been found significant inverse correlation in some studies [21,22] or no correlation [5]. A novel finding of this present study was that age was negatively correlated with fibre angles of the trunk extensor muscles (r = .40, p < 0.01). The spines of adults are normally able to withstand and absorb normal spinal forces even when the spines are subjected to sudden or unexpected changes as in falls and lifting. However, it may be argued that spines in older adults may not have the same ability due to changes in both the fibre angles and thoracic kyphosis as observed in this study. These changes in mechanics may be related to the occurrence of vertebral fractures in older adults’ spines which are osteoporotic [23].

As age was strongly associated with lumbar extensor muscle strength, the multiple linear regression analysis was performed separately for younger and older adults. From the stepwise regression analysis results, gender manifested as the most robust predictor of lumbar extensor strength, accounting for 53% and 21% of the variation in lumbar muscle strength of older and younger adults respectively. However, when considering the prediction of lumbar extensor muscle strength of older adults, age becomes another strong factor to be included. These results are expected, because a greater decline in trunk extensor muscle strength occurs from the fourth decade of life [4] and younger adults in this study were between the ages from 20 to 35. These results suggest that age affects lumbar extensor muscle strength in older but not younger adults.

**Figure 1:**
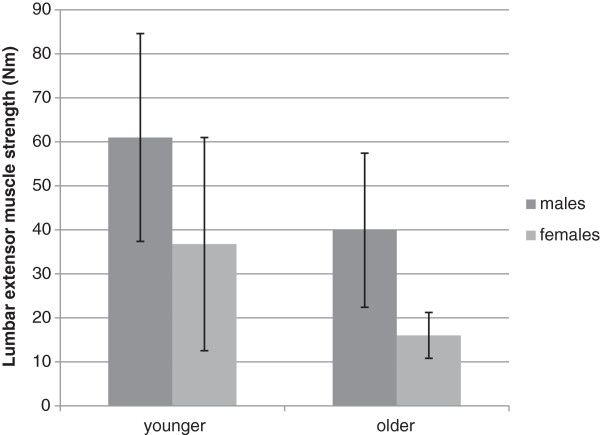
Lumbar extensor muscle strength in younger and older adults.

The decrease in trunk muscle strength is believed not to occur in a linear pattern with age. Moreover, it has been demonstrated that trunk muscle strength declines at a higher percentage in older adults [3,20]. The results from the regression analysis in the present study confirm this observation since in younger adults, gender appeared to be the only predictor for lumbar extensor muscle strength, while it was gender and age in older adults. The estimated rate of decline of lumbar extensor muscle isometric strength per decade from the sixth to eight decade in this study is approximately 40% and 81% in females and 21% and 41% in males. These results suggest that the rate of decline of lumbar extensor muscle strength in older women is approximately two times higher than that among older men. The results also demonstrate that there is a larger difference in lumbar extensor muscle strength between genders among older adults compared to younger adults (Figure 1).

From the regression analysis, thoracolumbar curvature and lumbar extensor muscle fibre angles did not emerge to be predictors of lumbar extensor strength. Although there was significant correlation of lumbar extensor muscle strength and fibre angles, it did not appear to be a strong predictor of strength**.** It can be deduced that some spine morphological factors are related to lumbar extensor muscle strength. However, it does not appear in the regression equation for both older and younger populations, probably because age or gender has accounted for such effects. Trunk extensor muscle strength has been reported to be based on the muscle area parameters such as cross sectional area and fat free mass [6,16]. Examination of the relationship between lumbar extensor muscle strength and a combination of the whole lumbar extensor muscle architectural parameters, including fascicle length and physiological cross sectional area that were not examined in this study could also possibly explain why lumbar extensor muscle fibre angles did not appear as a strong predictor.

The stepwise linear regression met the normal distribution and linearity assumptions. The adjusted R^2^ (coefficient of determination) were .17 and .61 (with two predictors) for the younger and older adults model respectively. These differences from the R^2^ are approximately 3%. Hence the lumbar moment strength prediction in this study can be generalised to the general population with a small variance in the equation results. The R^2^ in other back strength prediction models with younger participants were reported to be .39 [16] and .45 [6]. The lumbar strength prediction model of the older adults in this study showed a better lumbar strength predictability for older adults (R^2^ = .64, p < 0.001).

Future studies should consider including the other indices of muscle architectural properties such as fibre length to further understand the relationship. This study is limited to only two age groups, including a middle age group could have provided a data set that might help to demonstrate the relationships between some of the variables. Moreover, a longitudinal study could provide a definitive trend and the degree of the problem that can inform the rehabilitation team regarding at what stage of life intervention should be provided and would be beneficial. This is because there are many other factors besides ageing that influences muscle strength.

## Conclusion

In conclusion, significant correlations were found between age, thoracic kyphosis and fibre angles of the lumbar muscles and lumbar muscle strength; fibre angles of the lumbar extensors muscles with their strength. However, only age and gender appeared in the regression analysis in older adults. In the regression equation for younger adults, gender was the only predictor for lumbar extensor muscles. These results suggest that the decline in the lumbar extensor muscle strength might depend on different factors across the ages and different models are required for older adults.

Quantitative assessment of lumbar extensor muscle strength in older adults can be difficult and may be influenced by many factors. The regression analysis equation in the study could become a useful clinical tool after it has been refined through gathering of more normative data to predict lumbar extensor muscles strength, in situations where direct measurement of muscle strength would be inappropriate. This regression equation can also be used to compare normative data with the clinical population such as low back pain, osteoporosis and spinal deformities. Thus, it provides an estimate for the clinical judgment of the amount of lumbar extensor muscle deficit present.

## Competing interests

The authors declare that they have no competing interests.

## Authors’ contributions

All authors developed the protocol and interpreted the data. DKAS conducted data collection and data entry. DKAS wrote the manuscript and both RL and MB edited the manuscript. All authors contributed to and have approved the final manuscript.

## Pre-publication history

The pre-publication history for this paper can be accessed here:

http://www.biomedcentral.com/1471-2474/14/215/prepub

## References

[B1] Doherty TJ Physiology of aging. Invited review: aging and sarcopenia J Appl Physiol 2003 95 1717 1727 12970377 10.1152/japplphysiol.00347.2003

[B2] Frontera WR Hughes VA Fielding RA Fiatarone MN Willaim JE Roubenoff R Aging of skeletal muscle: a 12-yr longitudinal study J Appl Physiol 2000 88 1321 1326 10749826 10.1152/jappl.2000.88.4.1321

[B3] Limburg PJ Sinaki M Rogers JW Caskey PE Pierskalla BK A useful technique for measurement of back strength in osteoporotic and elderly patients Mayo Clin Proc 1991 66 39 44 10.1016/S0025-6196(12)61173-2 1824867

[B4] Sinaki M Nwaogwugwu NC Philips BE Mokri M Effects of gender, age and anthropometry on axial and appendicular muscle strength Am J Phys Med Rehabil 2001 80 5 330 338 10.1097/00002060-200105000-00002 11327554

[B5] Eagan MS Sedlock DA Kyphosis in active and sedentary postmenopausal women Med Sci Sports Exerc 2001 33 5 688 695 11323534 10.1097/00005768-200105000-00002

[B6] Keller A Johansen J Hellesnes J Brox JI Predictors of isokinetic back muscle strength in patients with low back pain Spine 1999 24 3 275 280 10.1097/00007632-199902010-00016 10025023

[B7] El Haber N Erbas B Hill KD Wark JD Relationship between age and measures of balance, strength and gait: linear and non-linear analyses Clin Sci (Lond) 2008 114 12 719 727 10.1042/CS20070301 18092948

[B8] Lauretani F Russo RC Bandinelli S Bartali B Cavazzini C Di Iorio Rantanen T Guralnik JM Ferucci L Age associated changes in skeletal muscles and their effect on mobility: an operational diagnosis of sarcopenia J Appl Physiol 2003 95 1851 1860 14555665 10.1152/japplphysiol.00246.2003

[B9] Rolland Y Lauwers-cances V Cesari M Vellas B Pahor M Grandjean H Physical performance measures as predictors of mortality in a cohort of community –dwelling older French women Eur J Epidemiol 2006 21 113 122 10.1007/s10654-005-5458-x 16518679

[B10] Sayer AA Syddal HE Martin HJ Dennison EM Roberts HC Cooper C Is grip strength associated with health –related quality of life findings from the Hertfordshire cohort study Age Ageing 2006 35 409 415 10.1093/ageing/afl024 16690636

[B11] Rantanen T Guralnik JM Foley D Masaki K Leveille S Curb JD White L Midlife hand grip strength as a predictor of old age disability J Am Med Assoc 1999 281 558 560 10.1001/jama.281.6.558 10022113

[B12] Hernandez ME Goldberg A Alexander NB Decreased muscle strength relates to self-reported stooping, crouching, or kneeling difficulty in older adults Phys Ther 2010 90 67 74 10.2522/ptj.20090035 19942678 PMC2802823

[B13] Singh DKA Bailey M Lee R Ageing modifies the fibre angles and biomechanical role of the lumbar extensor muscles Clin Biomech 2011 26 543 547 10.1016/j.clinbiomech.2011.02.002 21392870

[B14] Singh DKA Bailey M Lee R Biplanar measurement of thoracolumbar curvature in older adults using an electromagnetic tracking device Arch Phys Med Rehabil 2010 91 137 142 10.1016/j.apmr.2009.08.145 20103408

[B15] Singh DKA Bailey M Lee R Strength and fatigue of lumbar extensor muscles in older adults Muscle Nerve 2011 44 74 79 10.1002/mus.21998 21488056

[B16] Larivière C Gravel D Gragnon D Arsenault AB Loisel P Lepage Y Back strength cannot be predicted accurately from anthropometric measures in subjects with and without chronic low back pain Clin Biomech 2003 18 473 479 10.1016/S0268-0033(03)00026-3 12828894

[B17] Wang M Legar AB Dumas GA Prediction of back strength measurements in healthy females Clin Biomech 2005 20 685 692 10.1016/j.clinbiomech.2005.03.003 15905006

[B18] Pope MH Goh KL Magnusson ML Spine ergonomics Annu Rev Biomed Eng 2002 4 9 68 10.1146/annurev.bioeng.4.092101.122107 12117750

[B19] Pallant J SPSS survival manual: a step by step guide to data analysis using SPSS for windows (version 10) 2001 Open University Press

[B20] Sinaki M Khosla P Limburg PJ Rogers JW Murtaug PA Muscle strength in osteoporotic versus normal women Osteoporos Int 1993 3 8 12 8422518 10.1007/BF01623170

[B21] Mika A Is there any relationship between decrease in bone mineral density in women and deterioration in quality of life? Med Rehabil 2005 9 2 15 19

[B22] Sinaki M Brey RH Hughes CA Larson DR Kaufman KR Balance disorder and increased risk of falls in osteoporosis and kyphosis: significance of kyphotic posture and muscle strength Osteoporos Int 2005 16 1004 1010 10.1007/s00198-004-1791-2 15549266

[B23] Sinaki M Itoi E Rogers JM Bergstralh EJ Wahner H Correlation of back extensor strength with thoracic kyphosis and lumbar lordosis in estrogen-deficient women Am J Phys Med Rehabil 1996 75 5 370 374 10.1097/00002060-199609000-00013 8873705

